# Mixed‐methods evaluation of a continuing education approach to improving district hospital care for children in Lao PDR

**DOI:** 10.1111/tmi.13726

**Published:** 2022-02-13

**Authors:** Marianne Safe, Penelope Wittick, Khammanh Philaketh, Amphayvanh Manivong, Amy Gray

**Affiliations:** ^1^ Department of Paediatrics University of Melbourne Melbourne Australia; ^2^ Primary Health Care Program Office, Save the Children Luang Prabang Lao PDR; ^3^ Department of Paediatrics Mahosot Hospital Vientiane Lao PDR; ^4^ The Royal Children’s Hospital Melbourne Australia; ^5^ Murdoch Children’s Research Institute Melbourne Australia

**Keywords:** hospital care, paediatrics, public health, training

## Abstract

**Objective:**

To understand the impact of a multifaceted intervention on improving acute hospital care provided to children in two district hospitals in northern Lao PDR.

**Methods:**

We developed a continuing education intervention, which integrated separate program content using a common pool of facilitators and low‐fidelity simulation scenarios. Coaching was delivered over one year through two‐day hospital visits to each hospital six to eight weeks apart with visits incorporating feedback. A comparative case study was conducted between two hospital sites. Medical record abstraction from inpatient cases was performed at each visit. Focus groups and interviews with staff were conducted to understand perceived changes to case management.

**Results:**

Inpatient case management scores showed incremental improvement over time, from 50% at baseline to 80% at the end of one year at Hospital A and 52% to 97% at Hospital B. The key themes that emerged from the qualitative data from both hospitals were the value of the educational method and increased belief in capability. Hospital B showed more incremental and sustained improvement. Qualitative data revealed that the directors of Hospital B demonstrated modelling and behavioural reinforcement.

**Conclusion:**

Improving the quality of care in low‐resource settings is feasible. A hands‐on practical approach with repeated coaching visits reinforced by feedback can lead to behaviour change. Optimal impact requires harnessing leadership and motivation for change among health workers.

## INTRODUCTION

In low‐ and middle‐income countries,10 to 20% percent of sick children seen in primary care need referral to hospital [1, 2]. To improve child mortality in low‐resource settings, the quality of first referral level hospital care – the “critical link” – needs to be addressed [1, 3]. Resources to improve the quality of hospital care for children in settings with limited resources exist, including the Integrated Management of Childhood and Neonatal Illnesses (IMNCI), the WHO Pocketbook of Hospital Care for Children (‘the Pocketbook’), Early Essential Newborn Care (EENC), along with disease specific programs for HIV, tuberculosis, or other conditions [4, 5, 6]. The impact of these resources on improving quality of hospital care for children is influenced by how they are implemented and the context into which they are introduced. Often, they are introduced as parallel programs, with different approaches to how the content is taught or learnt despite targeting similar learners and relying upon a similar pool of facilitators. This approach to implementation potentially ignores the priorities for improvement for the learners and the health facility and limits the connections between programs (maintaining silos). Facilitators trained to deliver programs are potentially not learning transferrable skills, but how to teach IMNCI, the Pocketbook, or EENC in isolation. Instead, what if there was an established approach to continuing education or capacity building in a health facility, onto which new and evolving content could be scaffolded over time?

Lao People's Democratic Republic (Lao PDR or Laos) is a low middle‐income country in South East Asia. Child mortality remains among the highest in the region with the under‐five mortality rate at 45.5 per 1000 live births in 2019 [7].

In Laos, IMNCI implementation begun in 2003, Pocketbook in 2010, and EENC in 2013 [5, 8]. All rely on a limited number of Lao paediatricians in each province and their capacity (both in skills and time) is potentially rate‐limiting in scaling programs to the levels needed. Recognising these challenges, we designed a multifaceted intervention based on existing resources for child health and underpinned by a specific educational design that allowed for a consistent approach to teach or coach, integration of content, and audit and feedback on care. In this study, we aimed to pilot a multi‐faceted intervention to improve hospital care provided to children in two district hospitals. We aimed to measure observed and perceived changes to paediatric case management and determine the feasibility and acceptability of this intervention, alongside facility‐specific factors, which influenced the impact.

## METHODS

### Intervention method

**FIGURE 1:**
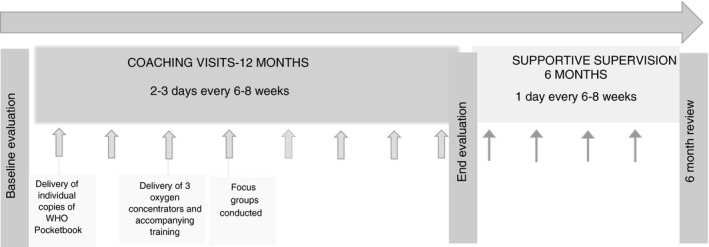
Schematic diagram of intervention implemented in Hospital A and Hospital B

We developed a multifaceted intervention to deliver separate program content in an integrated way, using a common pool of facilitators. We reviewed literature on educational method and instructional design, choosing an approach onto which content could be scaffolded over time. Flexible scenario‐based coaching modules were built based on Merrill's principles of instructional design [9]. These principles align well with Miller's pyramid of clinical competence and define the type of educational interventions required to move theoretical knowledge to clinical practice; from presentation of concepts to demonstration, practice, and feedback and reinforcement [10, 11]. Modules were initially based on the Pocketbook, EENC, and a previous Lao oxygen project content [4, 5, 6]. Modules for IMNCI have since been added. Coaching was delivered through two‐day visits to each hospital six to eight weeks apart for one year. The visits incorporated audit and feedback, as well as collaborative curriculum design – health staff were presented with the available education content and asked to prioritise the delivery of this content over each visit. At each visit, curricula requests were reviewed along with previously taught content for persistent deficits in understanding or practice. The year of coaching visits was followed by one day supportive supervision visits every six to eight weeks for a further six months (Figure 1).

The intervention is described in Table 1 using the Cochrane Effective Practice and Organisation of Care Review Group (EPOC) framework [12]. Two district hospitals (A and B) in Luang Prabang Province in northern Laos were identified as pilot study sites. Both were selected as initial targets because of their collaboration with a non‐government organisation who supported the implementation process and based on convenience—access from the provincial hospital. The same intervention was implemented in each hospital. Both hospitals have staff comprising of doctors, nurses, and medical assistants who share duties assessing and treating children. Common inpatient paediatric diagnoses are pneumonia, diarrhoea, dengue, and typhoid. Like many district hospitals in Laos, admission numbers are relatively low (approximately 300 children per year) due to issues of access, cost, and quality. Baseline characteristics of both hospitals are outlined in Table 2.

**TABLE 1:** The components of the intervention described according to the Cochrane Effective Practice and Organisation of Care Review Group (EPOC) framework

Feature of the intervention	Description
The key innovation	‐ Flexible delivery of coaching modules relating to hospital care for children in district hospitals in Lao PDR
The type of interventions used	‐ Distribution of educational materials (WHO Pocketbook) ‐ Distribution of equipment (oxygen concentrators) ‐ Small group educational meetings ‐ Bedside teaching including supervision ‐ Audit and feedback ‐ Local opinion leaders
The target group and incentives	‐ Staff working in district hospitals in Luang Prabang province treating children ‐ Coaching visits were on‐site, and staff were able to attend voluntarily as clinical and other duties allowed. There was no financial incentive to attend
The implementers	‐ Lao Paediatricians from Central and Provincial hospitals (considered local opinion leaders) ‐ International facilitators comprising Australian‐trained medical practitioners who have completed at least three years postgraduate training in paediatrics and completed their Royal Australian College of Physician exams ‐ Administrative and translation support from NGO
Intervention frequency and intensity	‐ Coaching visits occurred every six to eight weeks for 2 days at a time over a twelve month period ‐ Supervision visits were single‐day visits every six to eight week including audit, feedback, and targeted teaching if gaps were identified.
Feedback on performance	Assessment of case management of children from medical records: ‐ At baseline ‐ Verbally and in written form at each coaching visit ‐ After twelve months of the intervention at a formal meeting with district hospital staff, district hospital director, and district health officer director

**TABLE 2:** Preintervention characteristics of Hospital A and Hospital B from baseline Quality of Hospital Care Assessment

	Hospital A	Hospital B
Paediatric admissions year prior to intervention		
≤5 years old	203	186
>5 years old	101	90
Hospital facilities		
Number of Paediatric beds	6	No separate ward for children Children are admitted to general ward—12 beds including for adults
Number of newborn beds	1 x radiant warmer, 2 x open cots in maternity	1 open cot in maternity
Staffing (for whole hospital)		
Doctors	6	2
Nurses	16	6
Medical Assistants	5	5

### Research method

A comparative case study using mixed methods was conducted. The comparative case study approach was chosen to provide an in‐depth examination of the intervention over time in the two settings as part of a quality improvement approach before scaling the intervention to other sites [13]. Written permission to implement the intervention and to collect non‐identified data to understand its effectiveness was obtained from the director of each district hospital.

Medical record abstraction was performed with a standardised tool to collect information relating to the key steps in case management including history, examination, diagnosis, and appropriateness of treatment as recommended by the guidelines within the WHO Pocketbook. No identifying information regarding patients was collected.

The most recent ten inpatient admission records were reviewed each visit based on the principle that repeated measurement of small case numbers was appropriate for a quality improvement approach and that this would be most feasible in district hospitals with low admission numbers. Data from each hospital were entered and stored securely in a Microsoft Excel database, which was only accessible to the researchers.

The quality of case management was analysed in two ways and compared qualitatively between hospitals. First, the proportion of cases for which each case management step was correctly documented was calculated. The inpatient management scoring tool is outlined in Table 3. Second, the overall percentage of key correct case management steps was calculated to give an overall mean case management score for each visit. This approach aimed to provide data on both the overall performance of health workers, as well as detail pertaining to the areas in which case management improved.

**TABLE 3:** Quantitative research tool‐ inpatient case management score

Inpatient case management score	Scoring
What was the admission diagnosis?	‐
Was the diagnosis supported by the history/examination documented?	Y=1 N=0
Was weight recorded?	Y=1 N=0
Was height/length recorded?	Y=1 N=0
Was temperature documented on admission?	Y=1 N=0
Was HR documented on admission?	Y=1 N=0
Was respiratory rate documented on admission?	Y=1 N=0
Was SpO2 documented on admission?	Y=1 N=0
Was the treatment appropriate for the documented diagnosis/differential?	Y=1 N=0
Was any inappropriate treatment given?	Y=0 N=1
Were any drugs prescribed?	Y=0 N=1 Y=1 N=0 total =1
If yes, were all doses prescribed according to weight?	
Were any IV fluids given?	Y=0 N=1 Y=1 N=0 total =1
If yes, was the fluid rate based on weight?	
Was the child monitored adequately (twice per day or qid if severe)?	Y=1 N=0
Were all essential vital signs monitored?	Y=1 N=0
What was the discharge diagnosis?	‐
What was the discharge outcome?	‐
Total score/11 presented as percentage	
Additional case management scoring criteria for neonates aged <28 days	
Feeding: type and amount/frequency documented?	Y=1 N=0
If yes, appropriate for weight/age?	Y=1 N=0
For neonate with drowsiness or seizure—blood glucose documented or blood glucose bolus given?	Y=1 N=0
Pathological jaundice (jaundice to palms and soles or within first 24 hours of life ‐ was it referred?	Y=1 N=0
For every temperature <35.5 and >38 degrees—antibiotics given?	Y=1 N=0
Additional case management scoring criteria for acute respiratory infection	
Presence or absence of chest indrawing documented?	Y=1 N=0
Ability to drink documented?	Y=1 N=0
Conscious state documented?	Y=1 N=0
Illness classified appropriately? (URTI, pneumonia, severe pneumonia, bronchiolitis, asthma, other)	Y=1 N=0
Treatment appropriate for classification?	Y=1 N=0
Additional case management scoring criteria for diarrhoea	
Duration documented?	Y=1 N=0
Presence or absence of blood documented?	Y=1 N=0
Ability to drink documented?	Y=1 N=0
Skin pinch documented?	Y=1 N=0
Sunken eyes documented?	Y=1 N=0
Degree of dehydration documented?	Y=1 N=0
Is the documented dehydration appropriate/accurate?	Y=1 N=0
Does hydration plan match degree of dehydration?	Y=1 N=0
Is antibiotic prescribed/not prescribed appropriately?	Y=1 N=0

Focus groups and key informant interviews based on the Theoretical Domains Framework (TDF) were conducted approximately six months into the intervention to understand staff perspectives on barriers, facilitators, and perceived changes to care. The TDF is framework developed from synthesis of psychological theories as a vehicle to help apply theoretical approaches to interventions aimed at behaviour change. The qualitative research tool is outlined in Table 4.

**TABLE 4:** Qualitative Research Tool‐ domains explored, questions and prompts during interviews and focus groups

Domain	Question	Prompt
Knowledge and skills Current practice	What resources or materials do you use in your everyday work? The WHO Pocketbook? EENC Pocketguide? Oxygen concentrator? Job aids (ETAT/neonatal resuscitation posters?) None?	When used? How used?
Beliefs in capabilities Beliefs in consequences	What do you think about the different resources you have been given in through the project? (prompt for each one) WHO Pocketbook EENC Pocketguide Oxygen concentrators Hospital reporting program Do you feel that anything has changed in how you look after neonates and children in your hospital since the education visits from the project? Does anything prevent you from doing what you have learnt from the educational visits in your everyday work?	Do you have confidence in them? Why/why not? Are you confident in using them? Why/why not? What? Ask for specific examples. Ask in relation to each resource. What? Ask for specific examples. Ask in relation to each resource.
Social influences	Do you see other staff members using what they have learnt in their work? WHO Pocketbook EENC Pocketguide Hospital Reporting forms? Oxygen concentrators? Do other staff members encourage to use these resources? Do they prevent you?	Please give an example How?
Environmental context	Is there anything else that you need in your hospital to improve the care you give to children? Do the ideas or needs of patients and families prevent you from giving the care you want to patients? If you had the choice where and how would you want to learn about the Pocketbook, Newborn care, or other resources?	Staff Training Equipment Drugs Organisation (ask for specific details) Can you give an example? Your hospital At the province In central hospitals

All hospital staff present on the day of interview were invited to participate and none declined. Individuals interviews were performed if it was felt hierarchy may impact on response or for staff availability. Written and verbal explanations of the reason for the interview were provided and written consent obtained. Interviews were conducted by an international and Lao research team member together. The international researcher spoke Lao and had not participated in delivering the intervention. Questions were asked in English and translated into Lao language. Responses were in Lao land translated only where clarification was necessary. Discussions were audiotaped and then transcribed, deidentified, and translated into English. Data from focus groups and interviews were complemented by field notes made throughout the implementation process.

Interview transcripts were analysed using deductive content analysis to identify themes related to the TDF. The themes identified were reviewed within the research team in an iterative process to achieve agreement and compared between hospitals.

## RESULTS

### Impact on case management

A total of 173 inpatient case records were reviewed over nine visits at each hospital (86 at Hospital A between September 2015 and December 2016 and 87 at Hospital B between September 2016 and December 2017). The most common presentations were acute respiratory infections and diarrhoeal illnesses.

**FIGURE 2:**
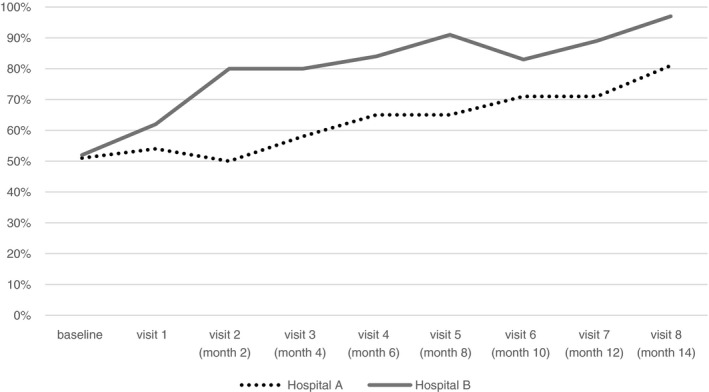
Inpatient case management scores in Hospital A and Hospital B during period of intervention

Inpatient case management scores showed incremental improvement over time, from 50% at baseline to 80% at the end of the full active intervention period at Hospital A and 52% to 97% at hospital B (Figure 2). Both hospitals demonstrated improvement in the proportion of cases in which weight was recorded (approximately 30% at baseline to over 90%) and alongside this, medication dosing according to weight (19% to 80% in Hospital A and 0% to 100% in Hospital B). The recording of vital signs (respiratory rate, heart rate, and temperature) on admission improved to 100% by the fifth visit in both hospitals.

Data collected six months after the initial year of coaching visits were analysed to understand the sustainability of change. Case management in both hospitals remained better than baseline with Hospital B demonstrating more sustained improvement (Hospital A 50% at baseline, 80% at end evaluation, and 70% after six months of supportive supervision; compared with Hospital B 52%, 97%, and 91%, respectively).

The prescription of inappropriate treatment showed minimal improvement over time in Hospital A but improved at Hospital B (Hospital A: 50% cases prescribed inappropriate treatment at baseline and 50% at end of full intervention, Hospital B: 40% at baseline and 20% at end evaluation). Inhaled salbutamol was commonly prescribed for patients under 12 months old with acute respiratory infection, which was likely to be bronchiolitis and have minimal effect. Mucolytics and antihistamines were prescribed for patients with acute respiratory infection, including to children under one month old where there is potential for harm.

Both hospitals showed initial improvement to intravenous fluid prescription, which was sustained in Hospital B but not in Hospital A. Through audit and feedback, Hospital A staff reported that there was a lack of agreement among staff regarding this and a large supply of hypotonic fluids available for use.

### Facilitators of hospital improvement

Key themes from the qualitative data were educational method, social influences, increased belief in capability, and perceived change. This educational method of linking theory to practice was different to the prior didactic teaching experienced by staff. It was described as an important enabling factor for change, *“they taught theories first…then showed how to do it in real life… they would support us to see patients and actually do it… it's quickly memorable” (Staff member 3*, *Hospital A)*.

Audit and feedback from medical records was an important intervention component as it allowed hospital staff to learn from examples of recent clinical practice. *“When we review [the medical records] we would be able to identify our mistakes and what we could improve… which we only know when they are showed” (Staff member 1*, *Hospital B)*.

The Pocketbook was viewed as a trusted guideline coming from respected external authority (WHO) and translated and adapted by local authority (Lao paediatricians). The method of all staff learning together despite role and seniority allowed for consistency of practice, creating an important social influence. It created a team approach and allowed for practice regulation between staff. *“Before the treatment seemed to be varied with different doctors*, *sometimes it was incorrect… in the end we could discuss and seek explanation and advised each other on up to date* / *new treatment recommendation” (Staff member 1*, *Hospital B)*.

Staff expressed increasing confidence in their capabilities and pride after gaining knowledge and skills to help their patients. Specific examples included treating severe pneumonia and managing premature babies who could not travel to the provincial hospital for treatment. *"I have used the oxygen concentrators and I am confident since it plays a part in helping patients’ recovery…*. *I have used it during neonatal resuscitation” (Staff member 4*, *Hospital B)*.

As reflected in the quantitative data, staff perceived there had been a significant improvement to care quality and provided various specific examples of change. These included classifying and treating diarrhoea according to severity and correct antibiotic therapy for acute respiratory infections. There was a change in culture to check medication and intravenous fluid prescriptions according to weight.

### Barriers to hospital improvement

Staff at both hospitals expressed that the main barrier to change to practice was lack of staff availability; “*One staff attend today's training*, *tomorrow a different staff member may attend the training*, *therefore*, *the training is not continuous” (Director*, *Hospital A)*.

Insufficient equipment or supplies was uncommonly raised as a barrier. In hospital A, one staff member perceived there was an ongoing lack of equipment for managing specific issues such as newborns and IV fluids in children. “…*there are obstacles when we are caring for preterm babies because we do not have an incubator… We do not have a syringe to adjust intravenous fluid for children*.*” (Staff member 3*, *Hospital A)*.

### Differences between hospitals

The quantitative measures of quality of care demonstrate greater improvement in Hospital B than Hospital A. When comparing qualitative data, Hospital B described themes of modelling and behavior reinforcement that were not apparent in Hospital A.

In Hospital B, the Director modelled attendance to all trainings. The Director spoke of how the training had empowered nursing staff to check the medical staff management, including himself, showing leadership to encourage the culture of regulation of practice between staff. *“Many staff can use the books… for example… I ordered Ampicillin… maybe I see some nurses they open (the book) because I think they want to know if I calculated the dose correctly” (Director*, *Hospital B)*.

Hospital B, led by the director, had provided incentives and reward for change, providing motivation. They discussed making someone responsible for oxygen concentrators every month and receiving a score for their care of the equipment, which was tied to a monetary incentive.

Toward the end of the initial year of the intervention period, staff at Hospital B had begun using the case management scoring guideline at morning handover to self‐assess the care of the patients overnight. This provided immediate feedback but also a culture of having a “safe space” to encourage audit and improvements, thus reinforcing change daily.

## DISCUSSION

We have demonstrated the success of a continuing education approach to improving quality of care in Lao district hospitals, which integrates the technical content of existing child health programs and responds to the learning needs of the health facility and staff. We were able to understand why there was behaviour change in two hospitals: due to the hands‐on practical approach with repeated visits over time.

Our study reflects aspects of the existing literature. Improved medication prescribing and measurement of vital signs were also reported with previous multifaceted interventions to improve hospital care for children [14, 15]. Previous studies in Laos, Kenya, and Kyrgyzstan also showed improvement to intravenous fluids prescription [14, 15, 16].

Both hospitals received the same intervention, in the same way with a greater sustained impact seen in one than another. It is useful to understand the commonalities and differences, to inform how future intervention may be enhanced. The Behaviour Change Wheel (BCW) is a tool created to design and validate interventions, provides on model for reflecting on these components [17]. This framework highlights key sources of behaviour including capability, opportunity, and motivation, and the interventions, which might influence them.

The coaching approach was clearly key in driving change in both hospitals by increasing staff capability. Staff were able to describe the educational approach accurately, despite it not being specifically articulated to them. Strategies such as small group education, supportive supervision, and audit and feedback have been identified as important enabling factors during previous interventions to improve hospital care for children [16, 18, 19, 20]. Our study added to this literature by clearly defining the educational intervention itself and the educational principles which were used, rather than just referring to the size of the group and its interactivity. Limited literature suggests that small group clinical simulation, even if it is low‐fidelity as in our intervention, does improve learning outcomes [21].

A review of literature to identify effective training approaches for health worker continuing professional education revealed that repetitive interventions were key to improving learning outcomes [22]. In addition, ensuring the content is relevant and realistic to practice was critical. Our outcomes support these conclusions. Furthermore, instead of the program defining the content for the learners, the learners drove the priorities for learning for each visit.

The barrier of incomplete training coverage was described by both hospitals and has been well reported previously. [20, 23, 24] Despite concerns regarding training coverage in both hospitals, there was improved clinical care. The collaborative curriculum design and ability for content to be delivered flexibly, and repeated if needed, is likely to have mitigated the problem of inconsistent staff presence. Furthermore, the benefits for training “in‐situ” at the hospital rather than removing a few staff for central training appeared to provide a unified approach, which outweighed the problem of staff availability.

In contrast, motivating factors were emphasized in Hospital B, where greater and more sustained changed was demonstrated, including modelling by leaders and enablement of staff at all levels. In Hospital A, Directors did not participate directly in any coaching visits. In contrast, Hospital B Directors participated actively in the preparation and delivery of the coaching visits and motivated their staff. These are potential future challenges for Hospital A but also possible targets for tailoring the intervention for this site. Leadership and modelling were identified as a barrier to uptake of paediatric guidelines in Kenya when not supportive of change [20, 23].

Hospital A described lack of equipment as a barrier to care, specifically relating to newborn care (incubators) and administration of intravenous fluids to neonatal and paediatric patients (infusion pump). This highlights a larger challenge in many low‐resource settings, including Laos, to implement Kangaroo Mother Care and Oral Rehydration Solution. In Laos, previous research highlighted parental expectations for intravenous fluids as a barrier to implementing diarrhoea guidelines [15, 25]. Kangaroo Mother Care is a more appropriate way to manage low birth weight neonates in this setting, instead of incubators; however, it is a complex intervention to adopt into health facilities and perceptions persist that equipment can offer better care [25, 26]. These barriers were not described in Hospital B where the guidelines were adapted in the environment despite having less resources than Hospital A, suggesting that leadership in change may overcome perceptions of equipment needs.

### Strengths and limitations

Members of the research team had roles in both implementation and quantitative evaluation of the intervention. They provided the coaching and collected case management scores using the audit tool to guide feedback, with this data also being used for evaluation. This strategy made our intervention and evaluation feasible, yet, we acknowledge potential for observer bias. To minimise this, the tool was highly structured with minimal user interpretation. To minimise potential positive reporting of intervention outcomes at the interviews, members of the research team who conducted interviews these were not directly involved in implementing the intervention. Trends in case management scores over time were only analysed at the end of the intervention period, alongside interview data to avoid influencing results.

Case management scores were gathered by medical record abstraction, based on the assumption that the clinicians documented the case management steps they performed. There can be a discrepancy between documentation and performance of tasks, so medical record data may not necessarily reflect an improvement in patient care, or true improvements in care may not be seen in the records [15]. Yet, the quantitative findings were supported by qualitative data as well as the observation of changes in practice as documented in the field notes. Furthermore, the evidence for change is based on only small numbers of case records at each visit. However, the sustained change over time and ability to triangulate the quantitative and qualitative data suggests that this change is real. Our methods provide a useful model of frequent data collection, using the same indicators to both monitor change and enable feedback which could be replicated elsewhere.

There were no other interventions occurring in the selected hospitals at the time, which would explain changes in care observed. Furthermore, longitudinal country monitoring of indicators over time in Laos have shown little change with respect to pneumonia and diarrhoea [27, 28]. There was one other intervention in the same province at the time focused on coaching on delivery and immediate post‐natal care, which would not contribute to the changes in care noted here.

This study provides understanding that the intervention is adaptable and sustainable in two different hospital contexts. Furthermore, it highlights factors at individual hospitals, including leadership and motivation, which may influence the degree of impact from any one intervention. In our current context of disrupted health systems, which need to maintain essential services models such as this that integrate separate program content and provide a consistent approach to upskilling workers at an individual hospital level are likely to be even more important.

The intervention has since been implemented in more district hospitals in this province and across Laos. Future studies will evaluate the ongoing impact as this approach is scaled up to other sites. The changes in quality of care were sustained at the six months following the full intervention. However, there is a need to understand the sustainability of the approach as it is scaled to other sites and the focused attention on these hospitals may lessen. In addition, there is a need to understand how factors such as leadership and motivation can be fostered or leveraged in other facilities.

## CONCLUSION

Our study has demonstrated sustained improvements in care at two district hospitals following a multifaceted intervention. Key reasons for this change were the participatory education approach and the health workers’ increased belief in their capability. In adopting this approach, we aim to create a learning system for health facilities, which can be built on over time as programs generate new content for health workers. Future studies should understand how to proactively harness leadership and motivation for the change among health workers.
